# Molecular Magnetic Resonance Imaging with Contrast Agents for Assessment of Inflammatory Bowel Disease: A Systematic Review

**DOI:** 10.1155/2020/4764985

**Published:** 2020-05-06

**Authors:** Yifan Luo, Chen Gao, Wujie Chen, Kefeng Zhou, Maosheng Xu

**Affiliations:** ^1^The First Clinical Medical College of Zhejiang Chinese Medical University, Hangzhou 310053, China; ^2^Department of Radiology, The First Affiliated Hospital of Zhejiang Chinese Medical University, Hangzhou 310006, China; ^3^Department of Radiology, Tongde Hospital of Zhejiang Province, Hangzhou 310012, China

## Abstract

**Background and Aims:**

Magnetic resonance imaging (MRI) has taken an important role in the diagnosis of inflammatory bowel diseases (IBD). In the wake of current advances in nanotechnology, the drug delivery industry has seen a surge of nanoparticles advertising high specificity in target imaging. Given the rapid development of the field, this review has assembled related articles to explore whether molecular contrast agents can improve the diagnostic capability on gastrointestinal imaging, especially for IBD.

**Methods:**

Relevant articles published between 1998 and 2018 from a literature search of PubMed and EMBASE were reviewed. Data extraction was performed on the studies' characteristics, experimental animals, modelling methods, nanoparticles type, magnetic resonance methods, and means of quantitative analysis.

**Results:**

A total of 8 studies were identified wherein the subjects were animals, and all studies employed MR equipment. One group utilized a perfluorocarbon solution and the other 7 groups used either magnetic nanoparticles or gadolinium- (Gd-) related nanoparticles for molecular contrast. With ultrasmall superparamagnetic iron oxide (USPIO) particles and Gd-related nanoparticles, signal enhancements were found in the mucosa or with focal lesion of IBD-related model in T1-weighted images (T1WI), whereas superparamagnetic iron oxide (SPIO) particles showed a signal decrease in the intestinal wall of the model in T1WI or T2-weighted images. The signal-to-noise ratio (SNR) was employed to analyze bowel intensity in 3 studies. And the percentage of normalized enhancement was used in 1 study for assessing the severity of inflammation.

**Conclusion:**

Molecular MRI with contrast agents can improve the early diagnosis of IBD and quantitate the severity of inflammation in experimental studies.

## 1. Introduction

Inflammatory bowel disease (IBD), including Crohn's disease (CD) and ulcerative colitis (UC), is a chronic, relapsing illness. The worldwide incidence of IBD has increased over the years, especially in western countries and newly industrialized countries [1, 2].

As a method for noninvasive examination, radiology takes an important role in the diagnoses of IBD by evaluating lesions in the intestinal wall. Compared with computed tomography (CT), magnetic resonance imaging (MRI) obtains high-quality images without harmful ionizing radiation, making it a more suitable modality for patients who require long-term follow-up, teenagers, and young adults. With a higher spatial resolution as well as various sequences, MRI is adapted to help diagnose IBD early and assess disease activity [3]. However, contrast-enhanced MRI sequences are often required for such diagnostic accuracy.

With the development of nanotechnology, new molecular probes have been developed for targeted imaging to evaluate lesions and surrounding tissues. The majority of the molecular contrast agents play a passive targeting role by being susceptible to macrophage clearance. Therefore, several studies rely on nanoparticles to monitor inflammation in the cardiovascular and nervous systems related to macrophage dysplasia [4–6]. On the other hand, active targeting probes can target track lesions via intracellular trapping. These modalities are mainly used in the evaluation of carcinomas and cardiovascular diseases [6]. It is worth noting that macrophage dysplasia also exists in intestinal inflammation and mucosal barrier damage in inflammatory regions increases permeability to some substances, providing a basis for the application of molecular imaging in the intestinal system [7, 8].

In this review, we assembled animal studies regarding magnetic resonance (MR) intestinal imaging using molecular contrast agents in the last twenty years. This review seeks to explore the advantages and characteristics of molecular imaging for the early diagnosis and quantitative assessment of IBD. We also assess the feasibility of molecular imaging for future clinical translation.

## 2. Materials and Methods

A systematic search of published studies in PubMed and EMBASE from 1998 to 2018 was conducted. Each database was searched for “magnetic resonance imaging” or “magnetic resonance” and “nanoparticle” or “nanoparticles” or “nanocrystalline material” or “nanocrystal” or “nanocrystals” and “gastrointestinal Tract” or “gastrointestinal tracts” or “GI tract” or “GI tracts” or “digestive tract” or “digestive tracts” or “digestive system”. No language restrictions were applied, and the references from selected articles were also scanned for additional relevant studies. The protocol for this review was registered on PROSPERO (http://www.crd.york.ac.uk/PROSPERO; CRD42019127236).

In the last two decades, molecular imaging was widely used to assess inflammation in the cardiovascular and nervous systems. However, literature regarding its use in intestinal imaging is scarce. Therefore, our group examined relevant literature without adding intestinal keywords to avoid excluding related articles.

**Figure 1:**
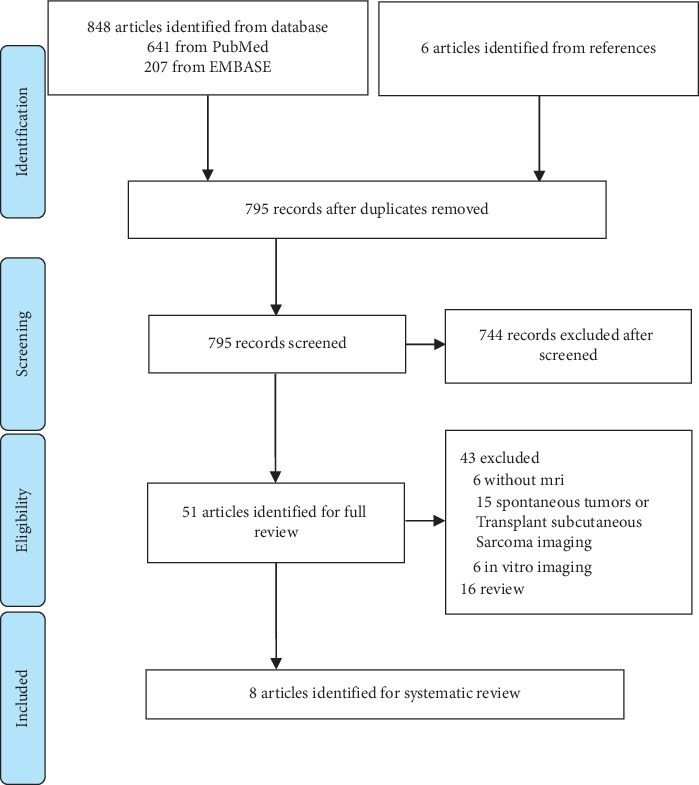
Flow diagram of the search strategy.

Titles and abstracts of potentially relevant studies were examined to determine whether they fulfilled the following inclusion criteria (see Figure 1): (1) studies must apply molecular materials to intestinal imaging and (2) use MR equipment. Articles that explored intestinal imaging ex vivo, in vitro, and in silico models were excluded. After reading the full text, articles which used either a spontaneous intestinal tumor model or only used molecular materials as the neutral contrast agent were also excluded. In the end, all articles either (1) assessed molecular probes designed for IBD diagnosis using healthy animals or (2) assessed molecular imaging for IBD or colitis-associated colorectal cancer models.

Data extraction was performed for animal species, sex, weight, age, the type of molecular contrast agent, administration method, absorption mechanism, MR equipment, MR sequence, and histological confirmation.

## 3. Results

Our literature search yielded 848 potentially relevant studies which used nanoparticles in MRI studies. There were 840 studies excluded for reasons listed in Figure 1. Characteristics of the 8 included studies were summarized in Tables 1 and 2.

### 3.1. Subjects

There were 8 studies matched the search criteria in which animal models of mice, rats, and rabbits were employed in articles 5, 2, and 1, respectively. Molecular MR imaging was facilitated in all 8 studies except for one, in which MRI was combined with single-photon emission computed tomography (SPECT/CT) [9]. Positive contrast agent was employed in 3 studies which showed abnormal strengthening in inflammation or colitis-associated dysplasia [10, 13, 14]. Another 2 groups used magnetic nanoparticles (MNPs) that showed signal intensity decreases in inflammatory lesions [9, 11]. Actively targeted nanoparticles were used in 2 studies, which can reach the lower digestive tract through oral administration and improve intestinal imaging [12, 15]. Perfluorocarbon (PFC) was adopted to tract macrophages in one study [16].

### 3.2. Experimental Animals

In the 5 murine studies, C57/B6, C56BL6, nude mice, and A/J mice were used, respectively [9, 11–13, 16]. The majority of studies used mice aged 5 to 8 weeks except for one study which used adult mice. Two studies adopted Lewis rats aged 8 to 12 weeks with an average weight of 220 to 270 g and Sprague Dawley rats with a 190 to 210 g body weight [10, 14]. The final study used a healthy rabbit at 2.7 kg in mass [15]. For modelling methods, three studies used healthy animals [12, 14, 15], two applied dextran sodium sulfate (DSS) to induce colitis in a mice model [9, 11], one used 2,4-dinitrobenzene sulfonic acid (DNBS) for colitis induction [10], and two groups treated mice with azoxymethane and DSS to generate a colitis-associated colorectal cancer model [13, 16].

### 3.3. Contrast Agents

PFC emulsions were used in 1 study [16] while other studies used nanoparticles. Four employed MNPs [9–12] and the other 3 adopted gadolinium- (Gd-) related nanoparticles [13–15].

#### 3.3.1. MNPs

In the MNP group, studies used ferumoxides (5 × 10^6^ superparamagnetic iron oxide (SPIO) nanoparticle-labeled macrophages per mice) [9], Nanotex (0.5 mg Fe/ml, 100 *μ*L per mice) [11], and casein-coated indocyanine green-loaded iron oxide nanoparticles (CN-ICG-IO) (10 mg Fe/kg) pertaining to SPIOs [12], while 1 study [10] used SHU 555C (5.6 mg Fe/kg) pertaining to USPIOs.

SHU 555C and Nanotex were taken up by the colon reticuloendothelial system (RES) to image inflammatory sites via intravenous administration [10, 11]. Wu et al.labelled macrophages with ferumoxide ex vivo and transfused the labelled cells back to the subject [9]. All studies located inflammatory sites by tracking the migration and distribution of macrophages.

In a separate study, CN-ICG-IO is encased by casein and modified with polyethylene glycol (PEG), which can be taken orally [12]. In another study, Nanotex combined with anti-inflammatory agents made it possible to integrate both diagnosis and treatment, creating a theranostic modality [11]. In histological confirmation, MNP groups used Prussian blue staining.

#### 3.3.2. Gd-Related Nanoparticles

Sun et al. [13] treated animals with Gd-loaded solid lipid nanoparticles (SLNs) (40 mg/ml, Gd-diethylenetriaminepentaacetic acid (Gd-DTPA) with a loading rate of ∼30%) by enema. The nanoparticles showed preferential accumulation in lesion areas due to colitis-associated, high-grade intraepithelial neoplasia, which creates higher permeability than healthy tissue. Cheng et al. [14] used Gd-DTPA-loaded chitosan nanoparticles (Gd in chitosan) (14.2 mg/ml, Gd-DTPA with a loading rate of 74.4%), and Perera et al. [15] used PEG-coated, Gd-incorporated, Prussian blue nanoparticles (Gd in PBNPs) (138.9 mg NPs/kg). Both of the aforementioned materials can be endocytosed to enhance colon mucosal uptake while the former is given by rectal administration and the latter is given by oral administration. In histological confirmation, Gd-loaded nanoparticles were combined with fluorescein isothiocyanate (FITC) to verify that material is absorbed by tissue [13].

#### 3.3.3. Perfluorocarbon Solution

Shin et al. [16] treated colitis-associated dysplasia mice with PFC (200 mg/ml, 200 *μ*l per mice) via tail vein iv injection. A ^19^F signal to noise was used to track labelled macrophages and assess the lesion area. In their study, inflammation in the descending colon was significantly increased compared to the ascending colon, while colitis-associated dysplasia and adenoma only appeared in descending colon. This finding proved that the occurrence of adenoma was closely related to that of inflammation.

### 3.4. MR Imaging Protocols

For the 5 murine studies, one group [11] used 7 T MRI equipment, one group [9] used 9.4 T MRI equipment, and one group [16] used 11.7 T MRI equipment while other groups [12, 13] used clinical 3.0 T or 3 T MRI equipment, and some studies [11, 16] mentioned the use of a 16–20 mm surface coil. In rat studies, one study [10] used a 2.4 T MRI and the other [14] used a clinical 3.0 T MRI equipment with a brain crossed coil. In the rabbit study, a clinical 1.5 T MRI was used [15].

For sequence selection, the PFC group [16] adopted a rapid acquisition with refocused echoes (RARE) sequence (repetition time (ms) (TR)/echo time (ms) (TE) = 1200/30 ms for ^1^H images and TR/TE = 1000/14 ms for ^19^F images). In the MNP group, ferumoxides [9] used a T1-weighted (T1W) black-blood spin-echo sequence (TR/TE, 600/8.6 ms), Nanotex [11] employed a T1W images by using a multislice multiecho sequence (TR/TE = 521/10.6 ms), CN-ICG-IO [12] used a T2-weighted (T2W) fast spin-echo sequence (TR/TE = 3600/86 ms), and SHU 555C [10] used a T1W 2D spin-echo sequence (TR/TE = 680/12.3 ms), T2W 2D turbo-spin-echo sequence (TR/TE = 3300/77.2 ms), and a T2^*∗*^-weighted 2D gradient-recalled echo sequence (TR/TE = 905/25.4 ms). For Gd-related nanoparticles, Gd-FITC-SLN [13] used a T1W spin-echo sequence (TR/TE = 550/15 ms) and Gd in chitosan [14] nanoparticles used a T1W spectral presaturation inversion recovery sequence (TR/TE = 456.47/14.5 ms) and T1W high-resolution isotropic volume examination sequence (TR/TE = 7.97/3.94 ms) was used for the for Gd-DTPA control group. Finally, PEG-coated Gd in PBNPs [15] used the 3D FLASH sequence.

### 3.5. Molecular Imaging and Improved Diagnostics

Gd-related contrast agents [13, 14] allowed significant enhancement in image quality for T1WI. Gd-based nanoparticles [13, 14] administered rectally showed a substantial increase in signal intensity along with the control group of Gd-DTPA. Nanoparticle-based gadolinium administered orally showed signal increases as well [15], through the addition of Prussian blue, and the aforementioned modality achieved notable increases in signal through endocytosis by intestinal epithelial cells.

Mucosal enhancement was observed in T1WI of rats with colitis after USPIO administration [10]. Meanwhile, deeper colon wall structures showed hyperintensity on unenhanced T2WI which presented as hypointensities after contrast enhancement. Two groups treated DSS-induced colitis with MNPs via intravenous injection [9, 11]; it was found that signal intensity decreased in T1WI. However, due to inherent artifacts or susceptibility artifacts, gradient-echo sequences, and T2^*∗*^, these sequences could not be used [9, 10]. An additional study demonstrated severe signal distortion through excessive material concentration [12].

The PFC group employed ParaVision software to combine a pseudocolor ^19^F signal with ^1^H imaging [16]. This allowed the tracking and assessment of intestinal inflammation through the distribution of the ^19^F signal.

Finally, three groups utilized the signal-to-noise ratio (SNR) to analyze the region of interest [10, 13, 16]. The SNR was calculated by dividing the signal intensity of the samples by the standard deviation of the background noise [17]. Finally, one group utilized the percentage normalized enhancement (%NENH) between the intestine and liver for quantitative evaluation [16].

## 4. Discussion

### 4.1. Molecular Contrast Agent Selection

MNPs are nanoparticles with iron and iron oxides as the main magnetic substances which have a higher relaxation degree and stronger effect on local magnetic fields than gadolinium-type molecular probes [18]. These include superparamagnetic iron oxide (SPIO) and ultrasmall superparamagnetic iron oxide (USPIO) particles. SPIOs such as ferucarbotran (SHU 555 A) are often more than 20 nm in diameter, with a core of Fe_3_O_4_ or Fe_2_O_3_ and modification with carbon, oxygen, or glucose. Due to the diameter of SPIO particles, they suffer from reticuloendothelial uptake in the liver and spleen. Meanwhile, the diameter of USPIOs is less than 20 nm, with an iron oxide core and dextran on the surface. Their small size permits them to enter lymphatic circulation and bone marrow [18, 19].

According to different chemical compositions, particle sizes, and surface properties, MNPs can be divided into T1W agents and T2W agents [20]. SPIO classifies as a T2W agent while USPIO has a greater T1 effect. The characteristics of positive enhancement make USPIO more suitable for diagnostic use. Like other organs and tissues, the MNP system has the ability to enter the colonic RES through circulation [9, 10]. Yet, signal distortion can negatively impact diagnosis if the local concentration is too high [10, 12].

Compared with the MNPs, gadolinium-like molecular probes do not have magnetic susceptibility artifacts. SLNs are a type of colloidal particle made from protein, dendrimer, or liposomes. Their sizes range between 50 and 1000 nm and they are biocompatible, biodegradable, and easily absorbed by the digestive tract [21].

**Figure 2:**
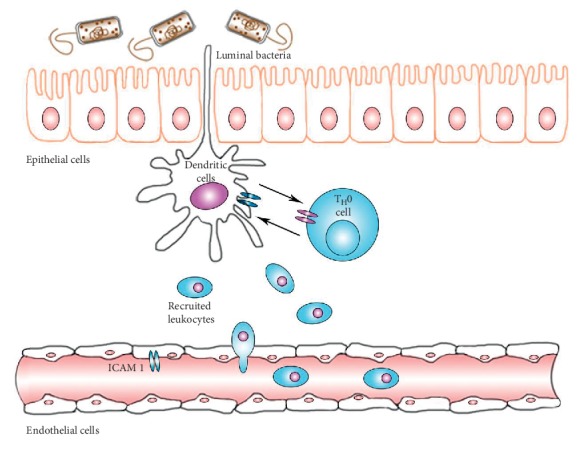
Leukocytes are recruited in the interstitial inflammatory response. Changes in the intestinal flora or a defective microbial clearing process may induce uncontrolled microbial growth and activation of proinflammatory processes [22]. There exists a mutual effect between dendritic cells (antigen-presenting cells) and naive T cells (T_H_0). Following activation, activated cells will differentiate and release various cytokines. Stimulated intestinal epithelial cells can secrete ICAM 1, which will recruit monocytes such as mononuclear cells, polymorphonuclear cells, and lymphocytes [7, 23, 24].

**Figure 3:**
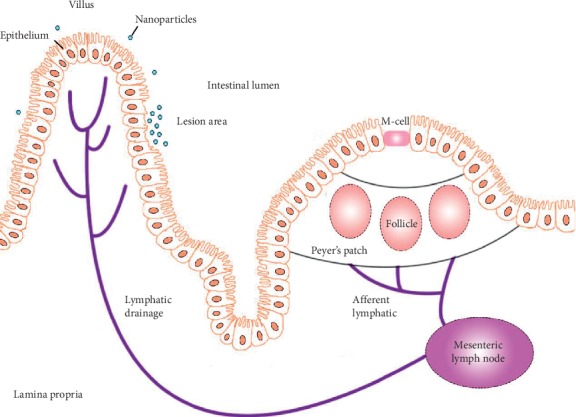
The absorption pathway of SLN. Lymphatic circulation plays an important role in SLN absorption [25]. Typically, most SLNs are either taken up by the M-cells overlying the lymphoid follicles, Peyer's patches, and circumambient lymphatic drainage, or transported via capillaries in direct or paracellular pathways [26–28]. However, in pathological situations, SLNs are primarily absorbed into the submucosal capillary network through the impaired colorectal mucosa. Furthermore, PEG-modified nanoparticles were more inclined to deposit at this lesion.

Leukocytes, such as macrophages, are commonly recruited in inflammatory reactions. Because of this, their migration can be traced using a molecular contrast agent (Figure 2). While most contrast agents require intravenous administration, SLNs are mainly absorbed via the intestinal tract (Figure 3). When given orally, absorption by the stomach accounts for less than 5% of total uptake [29]. It has been reported that about 50% of SLNs can be absorbed directly through intestinal mucosa or intercellularly, and the absorption correlates linearly within a certain concentration [21]. Specifically, M-cells overlying the lymphoid follicles and Peyer's patches may be the main ways SLNs enter the gut mucosa [25]. About 70% of absorbed SLNs were taken in circulation through the lymphatic system [21]. The remainder was transported directly through blood circulation, possibly via capillaries or intestinal epithelial cells by the paracellular route [26–28].

However, different conditions exist in pathological situations. Wu et al. [30] revealed that in DMH-treated mice, SLNs were primarily absorbed via carcinogenic colorectal mucosa and directly into the submucosal capillary network. Other studies have shown that the cell junction protein, zonula occludens 1, was imparted in the inflammatory state [8]. This led to expansions in the cell junctions and increased permeability, resulting in increased uptake in the carcinogenic regions. Using lymphatic transport, SLNs were able to avoid first past metabolism in the liver and increase absorption and efficacy.

Some controversy exists regarding the absorption of SLNs through the gut. One study demonstrated that detection of the SLN complex in intestinal epithelial cells was difficult when using a water-quenching, near-infrared (NIR) fluorescent probe [31]. Another experiment showed that SLNs with loaded drugs can pass through epithelial cells into intestinal capillaries, while lipids remain mostly in cells [32]. The characteristics of SLNs may be affected by different surface modifications as well as different manufacturing processes. Yet, multiple animal experiments have confirmed that SLNs can greatly improve the uptake and transport of the drugs loaded by intestinal epithelial cells [25, 33, 34].

For nanoparticle surface modifications, Gd in PBNPs utilized PEG to improve the sustained release and extend the therapeutic window [15]. Both animal experiments [35] and clinical studies [36] found that PEG-modified drugs were more inclined to deposit at the lesion, which may be related to the abnormal state of intestinal mucosa during inflammation. Additionally, it was found that Prussian blue can also be used in conjunction to enhance the drugs' penetration [20].

The use of ^19^F to trace cell migration in MRI imaging is based on the sensitivity of the ^19^F nucleus relative to ^1^H. Compared to metal ion contrast agents, PFC is nontoxic and has been applied for cell tracking in a clinical setting [37].

For the use of contrast agents, Wu et al. [9] reinfused labelled macrophages via tail vein intravenously. Three studies [10, 11, 16] adopted intravenous injection, two studies [13, 14] used rectal administration, and the other two studies [12, 15] used oral ingestion. Aiming to improve intestinal absorption, the nonintravenous groups used SLNs, chitosan, casein, or PEG to wrap the core material. Except for chitosan-based NPs, all other materials allow for oral administration. Compared to intravenous administration, the oral route is far less invasive with fewer side effects and improved compliance.

### 4.2. Imaging Optimization

For the development of the animal study, three key modalities were used. DSS induces inflammation in intestinal indiscriminately, while 2, 4, 6-trinitrobenzene sulfonic acid (TNBS) and DNBS can induce partial colorectal inflammation through topical administration. Therefore, TNBS and DNBS may provide an opportunity to observe the difference between normal tissue and inflamed intestinal tissue in the same segment.

The majority of studies used mice aged 5–8 weeks, which provided a better model for bowel inflammation. Acute models are suitable for analyzing short-term barrier changes and innate immune effects and flares. On the other hand, chronic models are able to illustrate the role of adaptive immunity, neoplasia, and tissue fibrosis [38]. While a rat or rabbit model has advantages for imaging due to their size, murine models can also achieve the same resolution if an appropriate coil is used to display the intestinal wall contour [13, 30].

Intestinal tract peristalsis and pneumatosis can induce nonspecific hypointensities and complicate the use of SPIO for the diagnosis of bowel diseases. To combat this issue, Wu et al. [9] treated mice with MnCl_2_ to delineate the intestinal contour, which allowed SPIO to distinguish the bowel wall from the intestinal cavity. Currently, oral administration of MnCl_2_ is approved by the Food and Drug Administration [39].

With gadolinium-like molecular probes, it has been reported that fluid-attenuated inversion recovery (FLAIR) is more sensitive to low-concentration gadolinium imaging [40]. In light of this, one study used FLAIR to collected high-resolution images in mice on a 1.5 T clinical scanner [30].

### 4.3. Future Expectations

Nanomaterials allow an unparalleled versatility in their ability to be loaded with therapeutic agents, genetic material, and various other compounds for treatment. For instance, Ohno et al. [41] treated murine colitis with nanoparticles loaded with curcumin. Zhang et al. [42] synthesized a redox-responsive b-cyclodextrin (OxbCD) and prepared Tempol- (TpL-) loaded OxbCD particles (TpL/OxbCD NPs) for the treatment of colitis in a murine model using oral administration. Separately, Huang et al. [33] prepared nucleic acid-loaded nanoparticles targeted to colonic macrophages. When given orally, these nanoparticles can be loaded with therapeutic agents that could be applied clinically. However, their studies were unable to monitor the activity level or drug distribution of intestinal inflammation in vivo.

Nanoparticles can also possess multiple functions when used as theranostic agents. Theranostic agents are agents which combine diagnosis and treatment, such as the combination of a contrast agent with a drug. Several studies have reported this method and found success in imaging using an in vivo imaging system or MRI [34, 43].

Despite the advantages of nanomedicine-based treatments, the major side effects primarily derive from the drug loaded within the carrier. For instance, recent studies have shown that gadolinium may exhibit deposition in the brain [44]. While concerning, no neurological symptoms have been reported to date [45]. Yet, gadolinium is contraindicated in patients with renal dysfunction due to the increased risk of nephrogenic fibrosis [44]. Finally, oral administration or enema administration has been shown to decrease side effects compared with iv injection, indicating the important role of the route of administration.

In spite of the findings listed herein, some limitations to the current study still exist. Firstly, only a few cases were recruited in each zoopery. Secondly, all research studies conducted were with animal studies, and further exploration is required to highlight the advantages that molecular MRI can have on the diagnosis of IBD for clinical trials. Thirdly, the sensitivity of MRI molecular imaging (micromolar) in comparison with positron emission tomography (picomolar) has a gap [46]. Through advances in molecular biology, nanoparticle-based targeted carriers, and bioengineering, the sensitivity of MRI for molecular imaging has risen. Undoubtedly, MRI imaging has proven itself as a highly versatile modality for the molecular imaging of inflammatory bowel disease, particularly for nanoscience-based delivery approaches.

## 5. Conclusions

With the development of a nanoparticle-based MR contrast agent, molecular MR imaging of IBD may improve and allow for (1) a comprehensive visual image of complex biological processes, demonstrating a new way to understand the molecular mechanisms of IBD; (2) monitoring of multiple processes at the same time; (3) improvement of early detection of IBD or metaplasia; and (4) monitoring of the mechanistic of drug delivery and efficacy in vivo. Furthermore, the advent of theranostics has been paving the way for combined modalities incorporating both diagnostics and therapy. When used in conjunction with MRI, nanomedicine-based therapies can greatly improve the diagnosis and treatment of IBD. The absorption characteristics of the intestinal tract provide a convenient basis for the oral administration of nanoparticles, which both improves patient compliance and decreases the incidence of adverse effects.

## Figures and Tables

**Table 1:** A summary of molecular MR contrast agents.

NPs	Material	Core	Coating	Animal model	Target	Characteristics
MNPs	Ferumoxides [9]	Fe_3_O_4_	Dextran	DSS-induced colitis mice model	Macrophage (ex vivo cell labelling)	Adept for cell tracking
	SHU 555C [10]	Iron oxide	Carboxydextran	DNBS-induced colitis Lewis rat model	RES	Positive contrast in MNPs
	Nanotex [11]	Fe_3_O_4_	SLN	DSS-induced colitis mice model	RES	Drug loaded
	CN-ICG-IO [12]	Iron oxide	Casein	Healthy nude mice	Gastrointestinal mucosal	Oral administration

Gd-related NPs	Gd-FITC-SLN [13]	Gd-DTPA	SLN	AOM- and DSS-induced mice model	Colitis-associated colorectal intraepithelial neoplasia	Absorbed via colon mucosal membrane functionality
	Gd in chitosan nanoparticles [14]	Gd-DTPA	Chitosan	Healthy Sprague Dawley rats	Gastrointestinal mucosal	Absorbed via colon mucosal membrane functionality
	PEG-coated Gd in PBNPs [15]	Gd in PBNPs	PEG	Healthy rabbit	Gastrointestinal mucosal	Oral administration, PBNP increases absorptivity

PFC	PFC emulsion [16]	—	—	AOM- and DSS-induced mice model	Macrophage	Metal ions free, adept for cell tracking

AOM, azoxymethane; CN-ICG-IO, casein-coated indocyanine green loaded iron oxide nanoparticle; DNBS, 2,4-dinitrobenzene sulfonic acid; DSS, dextran sodium sulfate; DTPA, diethylenetriaminepentaacetic acid; FITC, fluorescein isothiocyanate; Gd, gadolinium; MNPs, magnetic nanoparticles; NPs, nanoparticles; PBNPs, Prussian blue nanoparticles; PEG, polyethylene glycol; PFC, perfluorocarbon; RES, reticuloendothelial system; SLN, solid lipid nanoparticles.

**Table 2:** The applications of molecular contrast agents MRI in IBD-related model.

NPs	Material	Field strength	Sequences	Enhancement^a^	Administration	Procedures
MNPs	Ferumoxides [9]	9.4 T	T1WI	Intestinal wall T1WI (↓)	iv	Long enhancement sustained time, quantitative analysis
	SHU 555C [10]	2.4 T	T1WI, T2WI, T2^*∗*^	Mucosa T1WI (↑), deep colon wall T1WI (↓) &T2WI (↓)	iv	Quantitative analysis
	Nanotex [11]	7 T	T1WI	Colon wall T1WI (↓)	iv	Integration of diagnosis and treatment
	CN-ICG-IO [12]	3 T	T2WI	NA^b^	po	Oral administration, potential to monitor inflammation and targeted delivery

Gd-related NPs	Gd-FITC-SLN [13]	3.0 T	T1WI	Focal lesion T1WI (↑)	pr	Targeted imaging through intestinal absorption, low Gd deposition, quickly metabolized
	Gd in chitosan nanoparticles [14]	3.0 T	T1W	Mucosa T1WI (↑)^b^	pr	Targeted imaging through intestinal absorption, low Gd deposition
	PEG-coated Gd in PBNPs [15]	1.5 T	T1WI	NA^b^	po	Potential to monitor inflammation or targeted delivery, low Gd deposition, oral administration

PFC	PFC emulsion [16]	11.7 T	T1WI	Special^19^F signal in focal lesion	iv	Quantitative analysis; not affected by bowel movements or the existence of air pocket

CN-ICG-IO, casein-coated indocyanine green-loaded iron oxide nanoparticle; FITC, fluorescein isothiocyanate; Gd, gadolinium; iv, intravenously; MNPs, magnetic nanoparticles; NA, not available; NPs, nanoparticles; PBNPs, Prussian blue nanoparticles; PEG, polyethylene glycol; PFC, perfluorocarbon; po, per os; pr, per rectum; T1WI, T1-weighted image; T2WI, T2-weighted image. Notes: ^a^MNPs have both T1W and T2W effect, and these show the major effect with suitable dose in corresponding experiments; ^b^healthy animals were used in the experiment; (↑) signal increased; (↓) signal decreased.
